# Cortical neural synchronization in Alzheimer's and huntington's disease: Insights from resting‐state EEG

**DOI:** 10.1002/alz70855_106520

**Published:** 2025-12-24

**Authors:** Giuseppe Noce, Dharmendra Jakhar, Claudio Del Percio, Roberta Lizio, Susanna Lopez, Elena Salvatore, Ciccarelli Giuseppina, Marco Salvatore, Andrea Soricelli, Marina De Tommaso, Marianna Delussi, Claudio Babiloni

**Affiliations:** ^1^ IRCCS Synlab SDN, Naples, Italy; ^2^ Sapienza University of Rome, Rome, Italy; ^3^ University of Naples “Federico II”, Naples, Italy; ^4^ University of Bari Aldo Moro, Bari, Italy

## Abstract

**Background:**

Huntington's disease (HD) differs from Alzheimer's disease (AD) in its clinical presentation, particularly regarding vigilance, with notable motor, functional, and cognitive impairments that may be mirrored in resting‐state electroencephalographic (rsEEG) rhythms.

**Method:**

To evaluate this hypothesis, clinical and rsEEG data were gathered from age‐, sex‐, and education‐matched groups, including HD patients (*N* = 29), AD patients (*N* = 24), and healthy older participants (Nold, N = 29). EEG sources were computed using eLORETA software.

**Result:**

The findings are as follows: (1) Compared to the Nold participants, both AD and HD patients exhibited higher widespread delta source activities (with HD > AD); (2) HD patients with the most pronounced motor deficits showed very high delta and theta source activities across widespread cortical regions; (3) HD patients with the most severe cognitive deficits exhibited lower alpha and higher delta source activities in widespread cortical regions; and (4) HD patients with the most significant functional deficits displayed very high delta source activities in widespread cortical regions.

**Conclusion:**

These findings indicate that in HD patients during quiet wakefulness, disruptions in cortical neural synchronization at delta and alpha frequencies are distinctly linked to cognitive, motor, and functional impairments. This underscores a unique interaction between the cholinergic and dopaminergic systems, which likely modulates the generation of alpha and delta source activities.

